# Occurrence and Characteristics of Head Cysts in Children

**Published:** 2010-05-22

**Authors:** Noam Armon, Sivan Shamay, Alexander Maly, Alexander Margulis

**Affiliations:** ^a^Department of Plastic Surgery, Hadassah Medical Center, Jerusalem, Israel; ^b^Department of Oncology, Tel-Aviv Sorasky Medical Center, Tel Aviv, Israel; ^c^Department of Pathology, Hadassah Medical Center, Jerusalem, Israel

## Abstract

**Background:** Lumps arising in the head and neck constitute an important diagnostic category in children. As malignancy in this age group is relatively rare, lumps that are not due to inflammatory or infective causes often prove to be cysts. Correct diagnoses of the different subcutaneous lumps are often missed because of the lack of recognition or uncertainty with management. **Objectives:** The purpose of this study was to review the characteristics of most common pediatric cysts appearing in the head. **Methods:** A retrospective study was designed to review all the children treated for a cyst in the head during the 12-year period from 1995 to 2007. Study patients had a preoperative diagnosis of a cyst in the head, were younger than 11 years at the time of the diagnosis, and had received a surgical treatment for the condition. The pathological specimens were revised with an expert dermatopathologist, and the clinical data were statistically analyzed. **Results:** Of the 90 cysts, 53 were dermoid cysts (58.88%), 16 were diagnosed as pilomatrixomas (17.77%), 5 cysts were diagnosed as branchial cysts (5.55%), and 12 were diagnosed as epidermal cyst (13.33%). Male gender and younger age were significantly associated with correct diagnosis of dermoid cysts (*P* <.05). **Conclusions:** Ninety-five percent of the cysts fell in 1 of the 4 following categories: dermoid cyst, pilomatrixoma, epidermal cyst, and branchial arch cyst. Dermoid cysts were the most common facial cysts (60%). Correct preoperative diagnosis was more accurate in cases of dermoid and branchial cysts.

Pediatric dermatologic complaints are a common cause of outpatient physician visits. From cysts and warts to birthmarks and vascular anomalies, primary care physicians recognize many “lumps and bumps” in childhood. Correct diagnoses of the different subcutaneous lumps are often missed because of the lack of recognition or referred to plastic surgery for a second opinion because of uncertainty with management. Entities such as dermoid cysts, pilomatrixoma, and branchial anomalies often fall into this category. Even though the clinical and pathologic diagnosis of these skin lesions in children is well described in the literature, it is common to see erroneous diagnoses of “sebaceous cyst” on small children and “pillar cysts” on coronal or lambdoid dermoid cysts.

A retrospective study was designed to review all the pediatric patients treated for a cyst in the head by the senior author (A.M.) at the Hadassah Medical Center in Jerusalem during the 12-year period from 1995 to 2007. Study patients had a preoperative diagnosis of a cyst in the head, were younger than 11 years at the time of diagnosis, and had received a surgical treatment for the condition. The pathological specimens were then revised with an expert dermatopathologist, and the clinical data were statistically analyzed in respect to the various types of cysts present in the study group. To better define the different types of pediatric cysts, we reviewed our experience with the presentation, diagnosis, and treatment of the different types of pediatric cysts of the head.

## MATERIAL AND METHODS

Approval by the institutional review board at Hadassah Medical Center was obtained before beginning this study. Basic demographic information for each patient, including gender, age at the time of surgery, and the means by which the patient came to the attention of the surgeon, was recorded. The presenting history and physical examination, clinic notes, and radiological reports were reviewed. The preoperative diagnosis was based on history, clinical examination, and radiology reports. The postoperative diagnosis was determined by reviewing the histological slides with an expert dermatopathologist. The histological data were recorded and compared with the diagnosis and the initial histology report.

## RESULTS

Eighty-nine children met the criteria for inclusion. They underwent surgery for 90 cysts (one child had 2 cysts). The cysts were classified into 4 different categories: dermoid cyst, pilomatrixoma, branchial cyst, and epidermal cyst.

Fourteen children (15.5%) had preoperative imaging: 12 patients (13.3%) underwent computed tomography (CT) and 3 patients (3.3%) underwent ultrasonography (US). One patient had both CT and U/S performed prior to the surgery.

Only 1 patient presented with a history of infection (an abscess). The average age for presentation was 16 months for the dermoid cysts and 5.4 years for pilomatrixomas. Children with branchial cysts (5 patients), presented at the average age of 6.85 years.

The treatment was site dependent, with all lesions removed by direct excision and layered closure.

### Diagnosis

The preoperative diagnosis was based on the patient's history, physical examination, and radiographic studies. The pathology slides were reviewed by an expert dermatopathologist to determine the final diagnosis.

Of the 90 cysts, 53 (58.88%) were diagnosed as dermoid cysts, 16 (17.77%) were diagnosed as pilomatrixomas, 5 (5.55%) were diagnosed as branchial cysts, and 12 (13.33%) were diagnosed as epidermal cyst.

### Location

Of the 53 dermoid cysts, 47 (88.67%) were located at the lateral eyebrow, 2 (3.7%) were located in the medial eyebrow or at the nasal bridge, 2 (3.7%) were located in the midforehead, and 1 (1.8%) was located preauricularly. One cyst (1.8%) was located in the upper forehead above to the coronal suture. Of the dermoid cysts, 94% were located along the coronal suture, as opposed to only 35% of the nondermoid cysts (*P* <.01).

This difference in location also manifested as a significant predictor of the cyst type by the physician (*P* <.01).

In 91% of the boys with dermoid cysts, the cyst was located along the coronal suture. Only 33% of girls with dermoid cysts had a location along the coronal suture (*P* <.01).

Male gender and younger age were significantly associated with correct diagnosis of dermoid cysts (*P* <.05).

Of the 16 pilomatrixomas, 7 (43.75%) were located in the cheeks, 7 (43.75%) were located in the eyebrows, 1 cyst (6.25%) was located preauricularly, and 1 cyst (6.25%) was located on the nasal dorsum.

Of the 5 branchial cysts, 4 (80%) were located preauricularly and 1 (20%) was located postauricularly.

The 12 epidermal cysts were located as follows: 1 (8.3%) in the scalp, 2 (16.6%) in the cheek, 1 (8.3%) in the midforehead, 1 (8.3%) in the temporal area, 3 (25%) in the eyebrows, 1 (8.3%) preauricularly, and 3 (25%) postauricularly (Table 1).

### Correlation between the clinical and the pathological diagnosis

In 78% of the cases, the preoperative diagnosis correlated with the histopathological diagnosis.

Ninety-five percent of the dermoid cysts and 100% of the branchial arch cysts were diagnosed correctly prior to the surgery. On the other hand, only 36% of the pilomatrixomas and none of the epidermal cysts had a correct clinical preoperative diagnosis. Most of the pilomatrixomas were erroneously diagnosed clinically as dermoid cysts (27%), nonspecific cysts (27%), or sebaceous cysts (8%).

## DISCUSSION

Lumps arising in the head and neck constitute an important diagnostic category in children. As malignancy in this age group is relatively rare, lumps that are not due to inflammatory or infectious causes often prove to be cysts.^1^ Because of the varied types and locations of these cysts, patients may present to a wide variety of practitioners, including primary care physicians, pediatric surgeons, otolaryngologists, plastic surgeons, ophthalmologists, and neurosurgeons, all of whom need to be aware of the diagnostic techniques and treatment options.

The purpose of this study was to review the characteristics of the most common cysts appearing in the head region in pediatric patients. Ninety cases of cysts presenting in the head of children treated with surgical excision during 12-year period were subjected to retrospective analysis. A review of the clinical and pathological diagnoses in these cases revealed that 95% of the cysts fell into one of the 4 following categories: dermoid cyst, pilomatrixoma, epidermal cyst, and preauricular branchial remnants.

**Figure 1:**
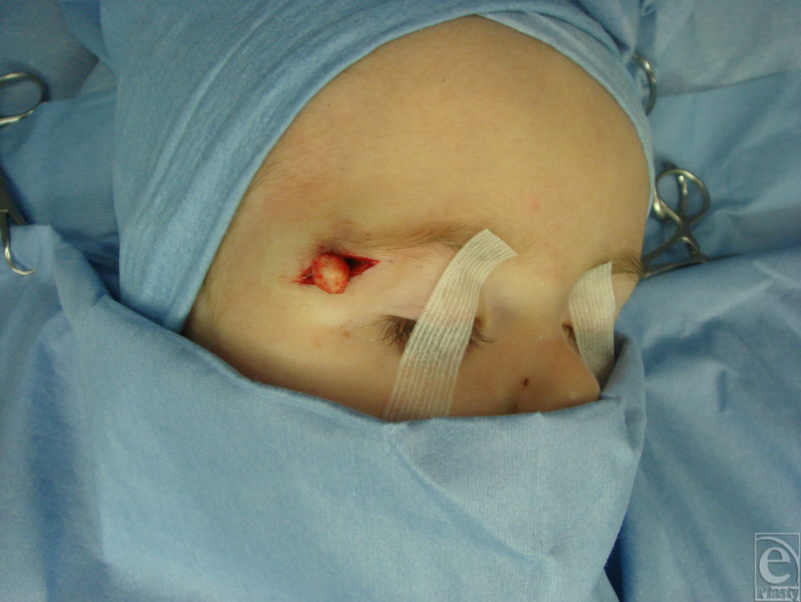
Dermoid cyst (intraoperative view).

A Dermoid cyst is a benign unusual neoplasm that is derived from both the ectoderm and the mesoderm (Fig 1). A keratinizing squamous epithelium is typically present together with dermal derivatives such as hair follicles, smooth muscle, sweat and sebaceous glands, and fibroadipose tissue. Although these neoplasms may be seen at birth, the age at presentation can vary widely, and sudden changes in size can make diagnosis more difficult.^2^ Approximately 7% of all dermoid cysts occur in the head, with the most commonly reported locations being periorbital, nasal, submental, and along the cranial sutures.^1^^-^6,7 Histologically, a dermoid cyst must contain 2 germ cell layers, and a pathologic confirmation is required to establish the diagnosis. Dermoid cysts are excised to establish a pathologic diagnosis, prevent subsequent infection, and ameliorate a cosmetically deforming lesion. Imaging studies are indicated when intracranial or intraorbital extensions are suspected.

**Figure 2:**
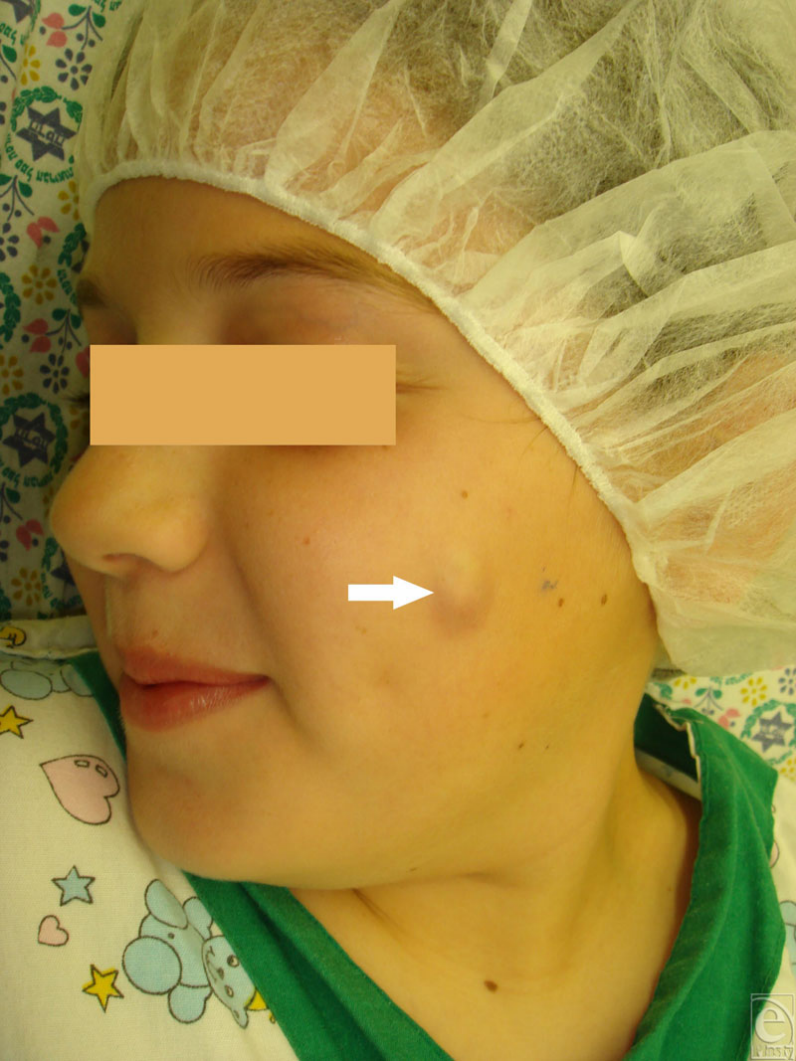
Pilomatrixoma arising in the left cheek.

Pilomatrixoma, also known as calcifying epithelioma of Malherbe, is a benign skin neoplasm that arises from hair follicle matrix cells (Fig 2). Clinically, it presents as a superficial hard mass with bluish discoloration of the overlying skin, which may become attenuated or ulcerated. Pilomatrixomas occur on the head, neck, and upper extremities and less frequently on the trunk and lower extremities. Surgical removal is generally curative, recurrence after complete excision is rare, and malignancy has rarely been reported.^8^ Pilomatrixomas are common and account for 1 of every 500 specimens submitted by surgeons. Despite their frequent occurrence, pilomatrixomas are often confused with other skin conditions.^8^ Pilomatrixomas can be distinguished from epidermoid and dermoid cysts by the presence of irregular nodules, which slide freely under the overlying skin.^8^ The overlying skin might have a red or blue hue.

Epidermoid cysts are firm, round, and mobile with normal overlying skin. In addition, epidermoid cysts often present among older patients (adolescents and adults).

Histologically, pilomatrixomas are sharply demarcated and contain basaltic cells and eosinophilic keratinized (shadow) cells. The proportions of these cellular components vary but the basaloid cells generally constitute the smaller component; in some cases, no basophilic cells are noted. These basaloid cells are fairly uniform in size, with round nuclei, small nucleoli, and delicate nuclear membranes. The shadow cells have distinctive cell borders and contain central unstained areas corresponding to the lost nuclei that are characteristic of pilomatrixomas. Mineralization in keratinized cells is a common feature. Another common finding for pilomatrixomas is granulomatous inflammation in areas of keratinization.

Branchial derivatives may take the form of cysts, sinuses, or cartilaginous remnants, and it is possible to identify the relevant branchial arch from the anatomic position. Strangely, although most remnants have usually been present since birth, branchial cysts most commonly present in adolescence or adulthood.^1^

### Preauricular and first branchial remnants

**Figure 3:**
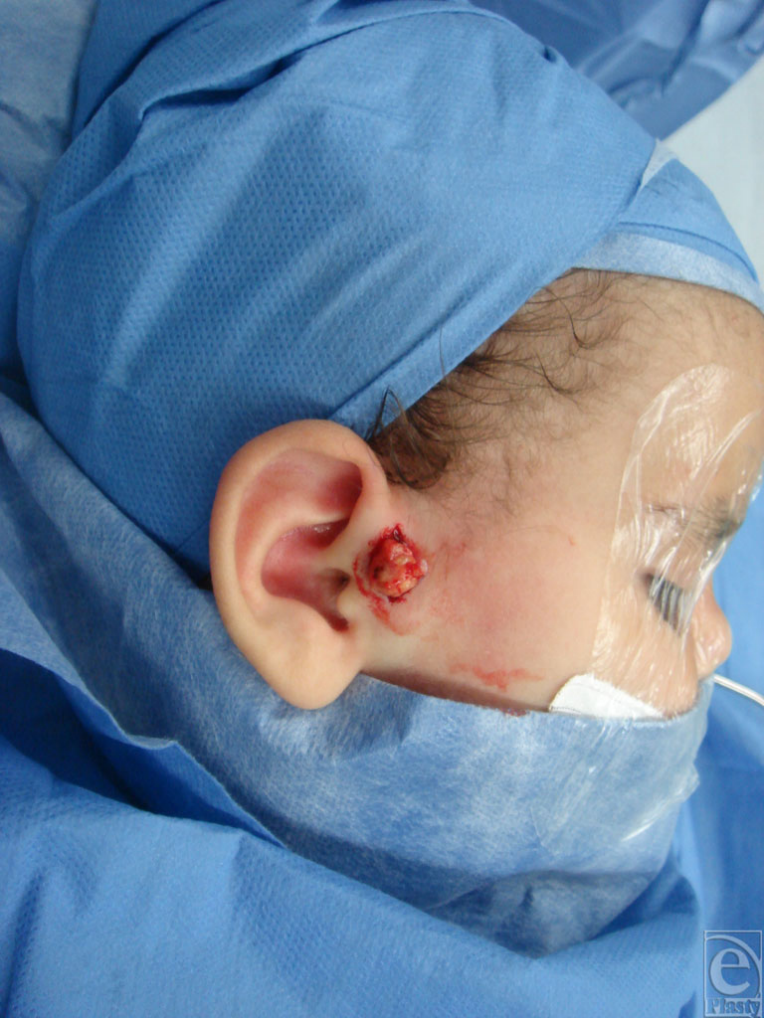
Preauricular branchial cyst (intraoperative views).

Small sinuses and cartilage remnants just in front of the ear are the commonest finding. Such preauricular pits may be blind but occasionally lead to a racemose collection of small cysts or to the cartilage of the helixn (Fig 3); otherwise, inconspicuous pits may present as an infection or an abscess in front of the ear. A true sinus or fistula from the first branchial arch is rare and has an opening just below the angle of the jaw along the uppermost border of the sternocleidomastoid. A communication with the external meatus may be identified during dissection.

### Location

The most common location for dermoid cysts was periorbital (92%), followed by the nasal bridge (4.76%) and the midforehead (4.76%). In this series, none of the dermoid cysts had intracranial or intraorbital extension.

The most common location of pilomatrixomas in the head was the cheek (45.5%) and the eyebrows (45.5%).

Eighty of branchial remnant cysts were located in the preauricular area.

### Symptoms

All children with dermoid cysts presented with a palpable mass. Other presentations included a change in the size of the mass, a fixed position, firm consistency, or cystic consistency. Only 1 patient (1.8%) had a history of local infection.

The most common presentation of pilomatrixomas was a hard, subcutaneous, slowly growing mass. No patients had a history of local infection.

The typical presentation of preauricular branchial cysts was a slowly growing mass in front of the tragus.

In this series, only one patient complained of pain in the area of the cyst.

### Imaging studies

Fourteen patients had preoperative imaging: CT in 12 patients and US in 3 patients (1 patient had both CT and U/S).

### Treatment

Treatment of all cysts was direct excision. For the preauricular branchial cysts, a window of helical cartilage was excised together and in continuity with the mass. The operative wounds were carefully closed in layers.

## CONCLUSIONS

In the differential diagnosis of pediatric head masses, cysts are common entities. The diagnosis is primarily based on history and physical examination findings. Radiographs, particularly CT scans, should be used only when intracranial or intraorbital extension is suspected. Surgical excision is the definitive treatment and is performed to prevent subsequent infections, establish pathological diagnosis, and ameliorate a cosmetically deforming lesion. Recurrence is unusual after complete excision.

- Ninety-five of the cysts fell in one of the 4 following categories: dermoid cyst, pilomatrixoma, epidermal cyst, and branchial arch cyst.
- Dermoid cysts were the most common facial cyst (60%).
- Imaging studies are indicated when intracranial or intraorbital extensions are suspected (midline lesions).
- Branchial cysts, though congenital, were operated at an oldest age (6.8 years).
- The most common location of dermoid cyst was periorbital.
- Correct preoperative diagnosis was more accurate in cases of dermoid and branchial cyst.
- The location manifested as a significant prediction of the cyst type by the physician.

## Figures and Tables

**Table 1:** Cysts type and location

Temporal region	Scalp	Nasal dorsum	Cheek	Postauricular	Preauricular	Forehead	Eyebrow	Number	Cyst type
…	…	…	…	…	1	3	49	53	Dermoid
…	…	1	7	…	1	…	7	16	Pilomatrixoma
…	…	…	…	1	4	…	…	5	Branchial arch
1	1	…	2	3	1	1	3	12	Epidermal
